# Detection and Grading of Gliomas Using a Novel Two-Phase Machine Learning Method Based on MRI Images

**DOI:** 10.3389/fnins.2021.650629

**Published:** 2021-05-14

**Authors:** Tao Chen, Feng Xiao, Zunpeng Yu, Mengxue Yuan, Haibo Xu, Long Lu

**Affiliations:** ^1^School of Information Technology, Shangqiu Normal University, Shangqiu, China; ^2^Department of Radiology, Zhongnan Hospital of Wuhan University, Wuhan, China; ^3^School of Information Management, Wuhan University, Wuhan, China; ^4^Division of Biomedical Informatics, Cincinnati Children’s Hospital Medical Center, Cincinnati, OH, United States; ^5^Department of Pediatrics, University of Cincinnati College of Medicine, Cincinnati, OH, United States

**Keywords:** glioma, detection, grading, gradient, classification, MRI

## Abstract

The early detection and grading of gliomas is important for treatment decision and assessment of prognosis. Over the last decade numerous automated computer analysis tools have been proposed, which can potentially lead to more reliable and reproducible brain tumor diagnostic procedures. In this paper, we used the gradient-based features extracted from structural magnetic resonance imaging (sMRI) images to depict the subtle changes within brains of patients with gliomas. Based on the gradient features, we proposed a novel two-phase classification framework for detection and grading of gliomas. In the first phase, the probability of each local feature being related to different types (e.g., diseased or healthy for detection, benign or malignant for grading) was calculated. Then the high-level feature representing the whole MRI image was generated by concatenating the membership probability of each local feature. In the second phase, the supervised classification algorithm was used to train a classifier based on the high-level features and patient labels of the training subjects. We applied this framework on the brain imaging data collected from Zhongnan Hospital of Wuhan University for glioma detection, and the public TCIA datasets including glioblastomas (WHO IV) and low-grade gliomas (WHO II and III) data for glioma grading. The experimental results showed that the gradient-based classification framework could be a promising tool for automatic diagnosis of brain tumors.

## Introduction

Gliomas are a group of primary brain tumors that arise from glial cells of the central nervous system (CNS). Traditionally, gliomas are classified by the World Health Organization (WHO) into four grades (from I to IV) depending on their histopathological features (Louis et al., 2007). However, the newest WHO classification of CNS tumors, published in 2016, combined both histopathological and genotypic features in the classification of these tumors (Louis et al., 2016). In this classification, WHO grade II and III are grouped under the low-grade glioma (LGG) category since they share common IDH mutations. Most of these tumors may also develop into the WHO grade IV glioblastoma (GBM) that has very high malignant degrees and poorer prognosis (Ohgaki and Kleihues, 2005). Compared to GBM, LGG has more optimistic outcomes with longer survival times (Brat et al., 2015). Therapeutic approaches are also different for these two groups of gliomas. Treatment for GBM normally includes surgical resection followed by radiotherapy with or without chemotherapy, while treatment for LGG is usually surgical resection followed by close observation (Woodworth et al., 2006). Hence, correctly grading of gliomas is very important for correct treatment. Conventionally, the determination of glioma grade depends on several histopathological features including mitotic activity, cytological atypia, neoangiogenesis, and tumor necrosis (Hsieh et al., 2017b). However, these features are not always easy to be recognized, and physicians may have different views about them, thus some misdiagnosis can still happen due to glioma heterogeneity or subjective judgments by physicians. Meanwhile, the surgery needs to resect some normal brain tissues, which may lead to sequelae, dysfunction or even functional loss after surgery.

With the rapid development of medical imaging technology, magnetic resonance imaging (MRI) is commonly used as a non-invasive tool to detect and determine the characteristics of brain tumors in clinic because it can provide a wide range of physiologically authoritative contrasts to recognize diverse tissues and enhances assessment of heterogeneous patterns of tissue compositions inside diffuse gliomas (Leach et al., 2005). Besides the typical sequences such as T1-weighted imaging (T1WI) and T2-weighted imaging (T2WI), other MRI techniques including diffusion-weighted imaging (DWI), MR spectroscopy (MRS), and perfusion-weighted imaging (PWI) can also be applied to discriminate between GBM and LGG (Provenzale et al., 2006; Tsougos et al., 2012; Svolos et al., 2013). Although a myriad of imaging data of gliomas has been produced every day worldwide, the detection and grade identification mainly depend on visual examination by experts, which is both time consuming and prone to errors. In recent years, artificial intelligence has made a huge impact on many aspects in human life including medicine and healthcare domain. Machine learning algorithms, the core techniques in artificial intelligence, can provide assistance for more automatic and objective diagnosis of brain tumors. Many algorithms can potentially discover the underlying subtle change patterns in patient brains by analyzing imaging data, which is sometimes difficult for humans to identify with eyes.

In MRI image analysis, feature extraction is a type of dimensionality reduction method that represents interesting parts of an image as informative features, facilitating the subsequent classification steps. For glioma detection and grading, traditional methods extracted hand-crafted image features and then trained machine learning models. Hsieh et al. (2017a) evaluated the malignancy of gliomas (GBM = 34, LGG = 73) using combination of global histogram moment features and local textural features, achieving an accuracy of 88% and an AUC of 0.89. Skogen et al. (2016) discriminated between low grade gliomas (grade II = 27) and high grade gliomas (grade III = 34 and grade IV = 34) using MRI textural features with different anatomical scales, achieving an AUC of 0.910. In recent years, deep learning methods such as convolutional neural networks (CNN) have shown the state-of-the-art performance in medical image analysis (Shen et al., 2017), including applications for tumor diagnosis. Yang et al. (2018) combined the deep learning with transfer learning for glioma grading (GBM = 61, LGG = 52) on conventional MRI images. AlexNet and GoogLeNet were fine-tuned from models that pre-trained on the ImageNet dataset, achieving the best test accuracy of 94.5%. Although these studies showed good performance on glioma grading, their experiments depended on manual selection of slices and ROIs by experienced neuroradiologists, which is time-consuming and error-prone. Zhuge et al. (2020) investigated fully automated methods for grading gliomas (GBM = 210, LGG = 105) by using deep CNN models including tumor segmentation and grade classification, achieving test accuracy of 97.1%. However, the process of distinguishing tumor boundaries from healthy cells is still a challenging task in the clinical routine. Some studies applied multistream deep learning method to obtain better performance. Ge et al. (2018) proposed a multistream deep CNN architecture for glioma grading followed by multi-modality MRI data fusion, achieving test accuracy of 90.87%. Ali et al. (2019) used the generative adversarial networks (GANs) for data augmentation and employed a multistream convolutional autoencoder (CAE) to extract multi-modality MRI features for classification of low/high grade gliomas, achieving test accuracy of 92.04%. Although deep learning methods have shown excellent performance in biomedical domains, their lack of interpretability still remains an issue, especially for clinical practice.

In this study, we propose a two-phase classification framework to distinguish patient from healthy controls or discriminate between GBM and LGG based on MRI images. Different from other studies, this framework analyzes all slices of a 3D MRI image without segmentation of brain tumors. In the first phase, we extract local features slice by slice using the Histogram of Oriented Gradients (HOG) algorithm. This algorithm helps to generate high-quality representations that depict image edge and texture. Zhu et al. (2015) proposed a multi-view learning method extracting both ROI features and HOG features from each MRI image for Alzheimer’s Disease diagnosis. Their method can help enhance disease status identification performance. Ghiassian et al. (2016) described a HOG-based learning algorithm that can produce effective classifiers for ADHD and autism. They applied the algorithm on two large public datasets and achieved good performance on both datasets. Since the histopathological characteristics of the two different grades of glioma influence the pixel intensity and spatial distribution within MRI images, we think that the HOG method may also help in the differentiation between GBM and LGG. We hypothesize that each local feature may be related to certain type, e.g., normal tissue or tumor tissue, with some membership probability. Then we combine the membership probability of each local feature into one high-level feature vector that is fed into the classifier trained in the second phase. By using this two-phase classification framework, we can identify whether tumors occur in the brain or predict the tumor grade.

## Materials and Methods

### Data Acquisition

In this study, we achieved two tasks in glioma diagnosis including glioma detection and glioma grading. We collected structural MRI (sMRI) data from patients with glioma and healthy controls in Zhongnan Hospital of Wuhan University. Because there is no grading information about these sMRI data, we used them in this study only for glioma detection task. We also downloaded the sMRI data from the Cancer Imaging Archive (TCIA) public repository. The data from TCIA only include images of different glioma grades, thus we used them in the glioma grading task. For convenience, we named these two datasets DS-Detect and DS-Grade, respectively, according to their different classification task.

The DS-Detect dataset contains 99 subjects including 62 patients with glioma and 37 healthy controls. Imaging was performed on a SIEMENS MAGNETOM Trio Tim 3.0T MRI Scanner. Whole brain coverage was obtained with 23 contiguous 6 mm axial slices (TR = 7,000 ms, TE = 94 ms, TI = 2,210 ms, FA = 130, matrix size = 464 × 512). The DS-Grade dataset includes 134 subjects among which 76 are diagnosed as GBM (grade IV), and 58 as LGG (grade II and III). Both datasets include three sMRI modalities: T1-weighted, T2-weighted, and T2-FLAIR. We chose T2-FLAIR modality since T2-FLAIR images are of higher-contrast and the high signal of tissue indicates the possible tumor growth.

### Image Preprocessing

As the first step of image preprocessing, the MRIcron tool was used to convert the original DICOM scans of an individual into a single NifTI image file. To solve the problem of non-standardized MRI intensity values among intra-patient and inter-patient acquisitions, we used the bias correction and Z-score normalization method, respectively. The scan bias correction algorithm implemented in SPM12 was used to minimizes MRI intensity inhomogeneity within a tissue region. To remove inter-patient intensity variability, we performed a Z-score normalization for each image, which normalize an image by simply subtracting the mean and dividing by the standard deviation of the whole brain, followed by clipping of the intensity value at [−4, 4] and a transformation to [0, 1]. To support comparison in a similar position at similar sizes, MRI image need to be spatial normalized. We used the spatial normalization procedure provided by SPM12 toolbox to register all MRI images to standard MNI space.

### Feature Extraction

Dalal and Triggs (2005) first applied HOG to pedestrian detection in static images and achieved higher performance than other local feature descriptors such as SIFT and HAAR (Dalal and Triggs, 2005). Since then HOG has been widely used in computer vision applications such as person and object detection or recognition (Qiang et al., 2006; Li et al., 2008; Yuan et al., 2009; Khan et al., 2010; Overett and Petersson, 2011; Kobayashi, 2013; Simo-Serra et al., 2013). The fundamental concept of HOG is that local object appearance and shape within an image can be described by the distribution of intensity gradients or edge directions. Here, we gave a brief introduction of HOG calculation process.

**FIGURE 1:**
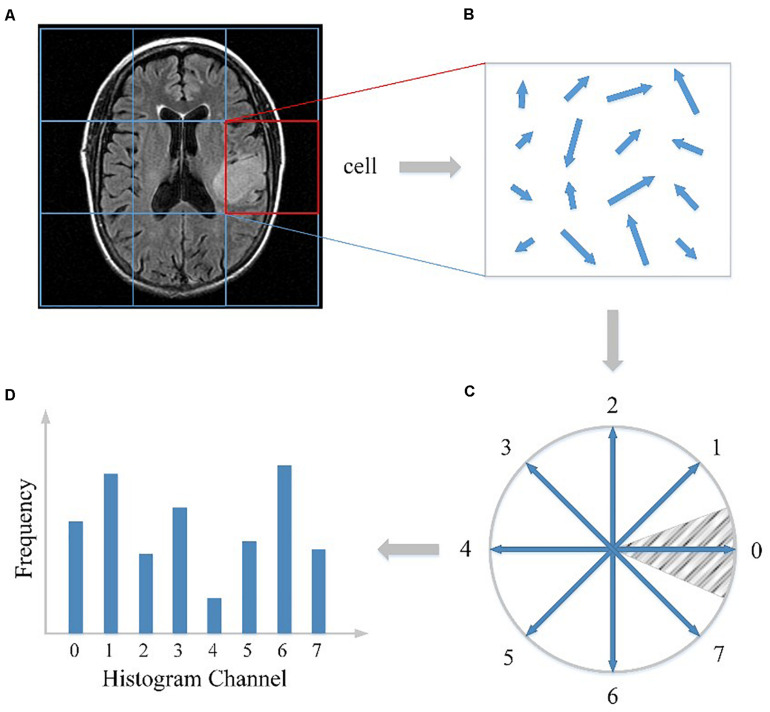
HOG feature extracted from the MRI image. **(A)** The MRI image divided into cells. **(B)** The gradient calculation result of one cell. **(C)** The partition scheme including 8 channels. **(D)** The cell histogram generated by counting the weighted number of pixels distributed in different direction channels.

First, as illustrated in Figure 1A, the whole MRI image was divided into a dense grid of uniformed spaced regions that is called cells in HOG’s terminology. The cell could be in any shape, but rectangular shape (R-HOG) and circular shape (C-HOG) are the most widely used ones. Due to better performance in the pedestrian detection experiments, we chose the R-HOG in this study. Secondly, the gradient of each pixel within the cell was calculated including magnitude and direction. Figure 1B shows the gradient calculation result of one 4 × 4 cell. Each arrow in the figure represents the corresponding pixel gradient – the arrow direction means gradient direction and the arrow length means gradient magnitude. Then a process called orientation binning was used to generate the cell histogram. The histogram is essentially a vector of N (e.g., 8) channels (bins) that are evenly spread over 0 to 360 degrees if the gradient is considered to be “signed.” Figure 1C shows a partition scheme including 8 channels. Each arrow represents the center direction of a channel. The channel 0 is illustrated as the shaded area, and so on. Thus, which channel is selected depends on the calculated pixel gradient direction. Furthermore, each pixel within the cell casts a weighted vote for its channel based on the gradient magnitude. Finally, a cell histogram was generated by counting the weighted number of pixels distributed in different direction channels as shown in Figure 1D. And the concatenation of all the cell histograms represents the feature descriptor of the MRI image. The histogram may be contrast-normalized to improve accuracy and reduce effect of changes of illumination and shadowing (Dalal and Triggs, 2005).

### Two-Phase Classification Framework

In traditional HOG application like pedestrian detection, the HOG feature vector of each cell is concatenated into one high-dimensional vector representing the 2D image. However, since the preprocessed MRI image is a 3D NifTI format, we need to extract the HOG feature from each slice then concatenate all the HOG features to form a representation of the whole 3D image. The dimension of the final HOG feature vector thus is quite high, which may cause the “curse of dimensionality” problem.

To address this, we proposed a two-phase classification framework that could transform the low-level gradient features into high-level semantic features. In the first classification phase, we calculated the HOG feature of every cell in an MRI image from the first slice to the last slice. Then instead of directly concatenating these local HOG features into one high-dimensional feature vector, we analyzed these local features independently and then integrated the analyzing results for further process. We have proposed this “local to whole” approach in a previous study that used SIFT features to diagnose the neurological diseases (Chen et al., 2014). Specifically, we first transformed each HOG feature into one real number, which indicates the probability of each cell relating to one specific type, and then these real numbers were concatenated to form a compact representation of the whole MRI image. This transformation from original HOG feature to cell type feature can reduce the dimensionality of feature space, thus alleviating the impact of overfitting problem. In the second classification phase, the new representation of brain images in the training set were used to train a classifier to detect gliomas from brains or distinguish between different grades. We take the glioma grading as example to illustrate the overall two-phase classification framework.

**FIGURE 2:**
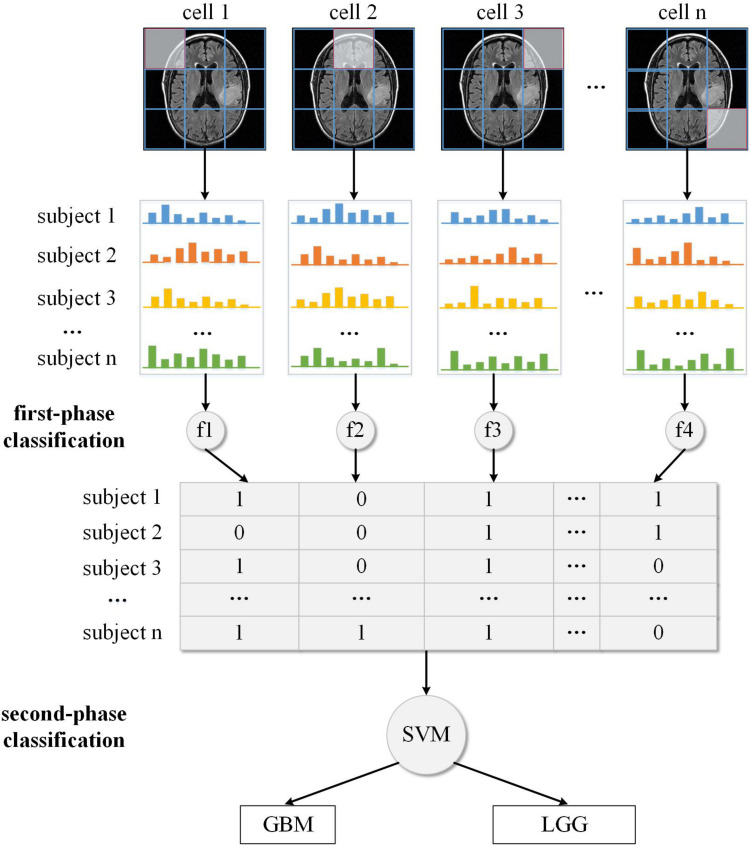
The gradient-based two-phase classification framework.

Some studies about brain tumors depend on experienced neuroradiologists to select the most representative slices from the image scan, and delineate the tumor contour manually as ROI for feature analysis (Skogen et al., 2016; Hsieh et al., 2017a). However, the tumor location can be anywhere in the brain, thus such ROI process may be labor-intensive and time-consuming, which is not appropriate for clinical practice. In this study, we designed an automatic pipeline to analyze the image features without manual or semi-manual ROI delineation. Firstly, the whole 3D sMRI image was sliced into a series of 2D images along the scan orientation. Then for every slice, the feature descriptor of each cell was extracted using the HOG algorithm. This feature extraction process is fully automatic and can cover the entire brain. For the sake of convenient illustration, only the slice with the largest axial cross-section of the tumor was used as an example in Figure 2. Suppose a 3D sMRI image includes *m* slices and each slice is divided into *n* cells, then a total of *m × n* cells are required to be analyzed for one brain.

After we applied the HOG algorithm to the sMRI image, each cell was represented by its local HOG feature. We analyzed each feature independently and supposed it to be related to different types. Because in the MRI preprocessing phase the MRI images were already aligned to the standard brain template, those HOG features that were extracted from the same location of different brains could be compared based on their gradient histogram, and then their respective feature type be identified as the Figure 2 illustrated.

Since the exact label of the whole brain rather than the type of each local brain region is known, we applied unsupervised clustering methods to discriminate the type of each HOG feature. In machine learning, a clustering method is used to determine a classification of *n* subjects into *K* discrete classes (*K < < n*) for a given dataset *X* = {*x_1_, x_2_, x_3_, …, x_*n*_*}, with each subject *x_*i*_, i = 1, 2, 3, …,n* characterized by m features, *xi* = (*x_*i1*_, x_*i2*_, x_*i3*_, …, x_*im*_*). The main objective of clustering is to discover and describe the underlying structure in the data. There are two kinds of clustering methods including “hard clustering” that exclusively assigns each subject to a single cluster, and “soft clustering” that is flexible and allows each subject to be assigned to more than one cluster. K-means, one of the most widely used hard clustering methods, partitions the subjects into k clusters given the data points and k number of centroids in an automated fashion. As a variation of the K-means algorithm, the fuzzy C-means algorithm is based on fuzzy logic principles and assigns each subject a possibility in each cluster center from 0 to 100 percent. In this study, we used the fuzzy C-means algorithm to calculate the possibility of each HOG feature being related to diseased/healthy status or GBM/LGG status. The reason we used fuzzy clustering method is that the boundary between these different types of image features is usually ambiguous, e.g., some cells may be located in both malignant and benign brain regions. So, if only using traditional hard-thresholding clustering method like K-means to classify the feature without considering the ambiguity, the classification result can not reflect the actual grouping complexity of the brain features thus leading to reduced classification performance. In Figure 2, the decimal numbers like 0.4, 0.6 are the probability of local HOG feature being classified into GBM-related cluster (or LGG-related cluster). For the two datasets, we have tried different *K* values, and found *K* = 2 is the best value. Furthermore, the clustering process also generated centers of the two clusters that can be used to predict to which cluster the local features of new unknown subject belongs based on the nearest centroid classification method.

The local feature clustering result only represents the status of individual brain regions, while the status combination of all brain regions is more significant for describing the underlying pattern of the whole brain. Thus for every subject, we concatenated the fuzzy labels of each feature from the first cell to the last cell across all the MRI slices as shown in Figure 2. Instead of directly concatenating original HOG feature of each cell, we used the clustering method to transform each HOG feature to another high-level feature that indicates the status of the corresponding brain region (e.g., 60% for malignant). This feature transformation can reduce data dimensionality and alleviate the problem of overfitting. As a new representation of each subject’s brain image, the compact probability vector was used as the input feature to a Support Vector Machine (SVM) discriminative classifier. In machine learning, SVM is a supervised learning algorithm that outputs an optimal hyperplane which separates the data samples into two classes. And it has been proved highly successful in solving a wide range of pattern recognition and computer vision problems such as text detection and image classification, also in the classification of MRI data (Magnin et al., 2009; Zacharaki et al., 2009; Ecker et al., 2010). If the data is not linearly separable in the original feature space, SVM can efficiently performs a non-linear classification using a so-called kernel function, implicitly mapping their inputs into high-dimensional feature spaces to achieve separability.

The above steps described the whole training process of the two-phase classification framework. This training process produced two classifiers, the nearest centroid for local brain regions and SVM for the whole brain image. Then we can apply these classifiers to unknown brain samples in the test dataset. Like the training process, the test brain sample was also divided into local regions and the HOG feature was extracted for each brain region. These local features were first identified to be related to different types using the nearest centroid classifier trained in the first phase. Then the classification results of all the individual features were combined and fed to the SVM classifier trained on the second phase to predict the final result of the test brain sample, e.g., whether the brain contains gliomas or what grade the glioma is.

## Results

To evaluate the performance of our two-phase classification framework and solve the problem of the imbalanced datasets, the stratified 10-fold cross-validation (CV) method was applied to train the models. The stratified method can preserve the proportion of positive to negative samples in each fold to match the original distribution in the whole dataset. Furthermore, the variance of the model will decrease by performing several random runs, in each of which all samples are first randomly shuffled and then split into a pair of train and test sets. Since we extracted HOG features slice by slice and combined their clustered results into one feature vector representing the whole 3D MRI image, our random data shuffling for CV is subject-separated.

**FIGURE 3:**
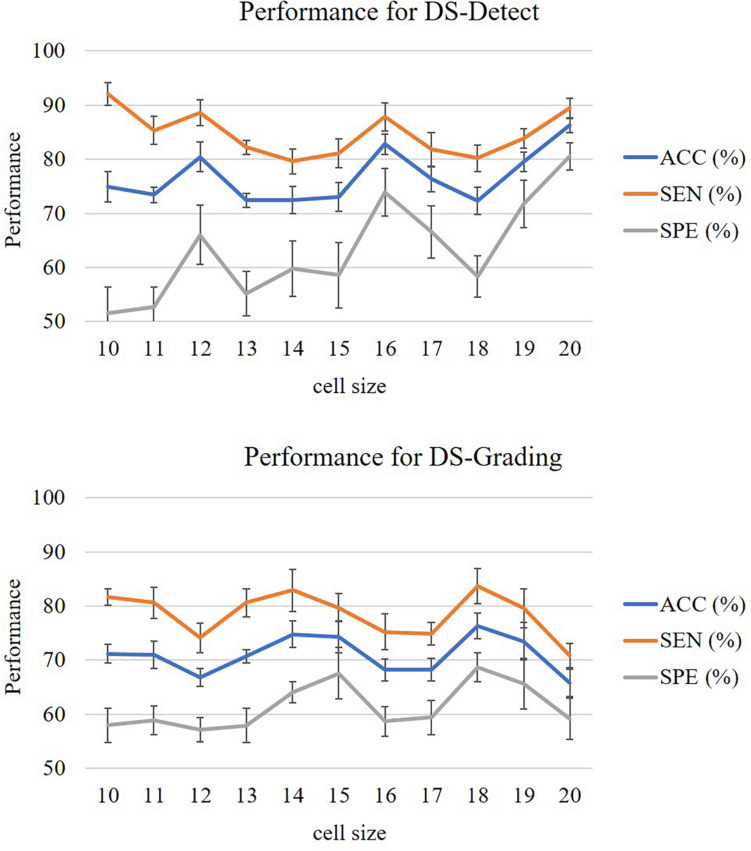
Performance of glioma detection and glioma grading.

In this study, we evaluated the performance of our proposed two-phase classification model using the following measurements: accuracy (ACC), sensitivity (SEN), specificity (SPE), and area under curve (AUC). These measurements can be calculated from the classification confusion matrix. Here, the accuracy is defined as the ratio of correctly classified subjects over all subjects. The sensitivity is the ratio of correctly classified subjects with glioma over all subjects with glioma, and the specificity is the ratio of correctly classified subjects without glioma over all subjects without glioma. The AUC refers the area under the receiver operating characteristic (ROC) curve. The larger AUC value means better model performance. For our unbalanced dataset, the AUC metric is especially useful to assess the overall performance of the model. In addition, the cell size is a parameter that will affect the performance of the model. In the experiment, we assigned the cell size with value from 10 to 20 and calculated the above measurements, respectively. In this study, we accomplished two classification tasks including glioma detection and glioma grading using our proposed gradient-based two-phase classification framework. Figure 3 shows the performance of these two classification tasks.

The measurements from Figure 3 were calculated after 10 random runs of stratified 10-fold cross-validation for each cell size. The figures showed that the performance for glioma detection outperformed that for glioma grading. The reason lies in that glioma detection is just to identify the occupying effect in the patient brain, while glioma grading intends to distinguish different morphological pattern between high grade and lower grade, thus the detection task seems easier than the grading task.

**FIGURE 4:**
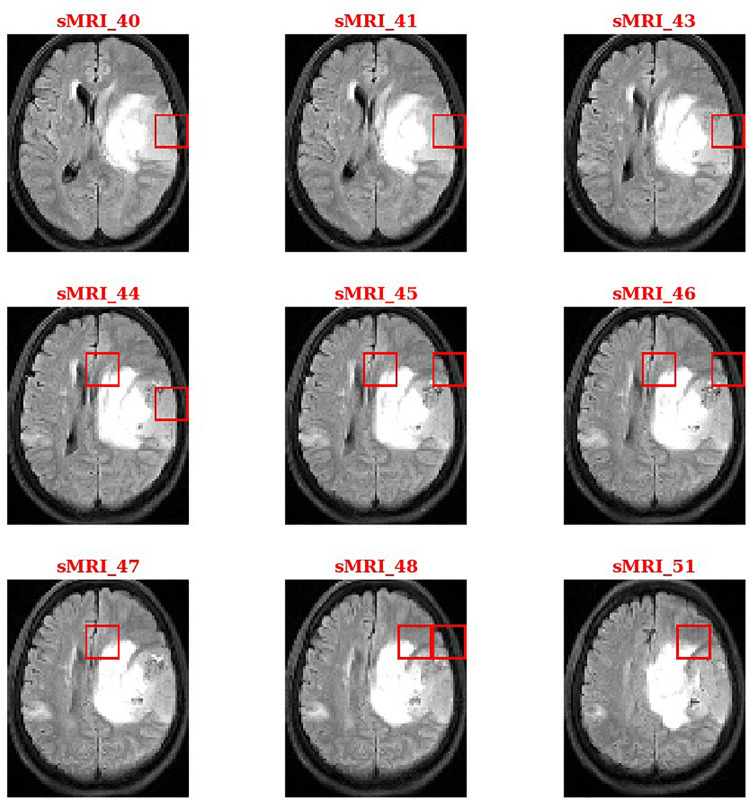
Discriminant features related to brain tumors.

During the second phase in SVM training, we obtained a weight vector that indicates the direction along which the two classes of subjects differ most. This vector can be used to identify and localize the most discriminant HOG features that account for case-control separation. By sorting the weights in descending order or setting a threshold value, we identified the brain regions that most likely related to tumors. Figure 4 shows the thresholding results for a patient with glioma.

The baseline method for HOG is that extracting the local HOG features and then directly concatenating them into one feature vector as representation of the whole image. In the experiment, we compared the performance between the baseline method and our proposed method, which transformed regional HOG features into high-level features, for each glioma diagnostic task. We also used the stratified 10-fold cross-validation strategy for the baseline method. Tables 1, 2 shows the performance of the two methods. The cell size in each table means the optimal parameter to obtain the best model performance.

**TABLE 1:** Comparison of the glioma detection performance evaluated by 10-fold cross-validation between the baseline concatenating HOG method and our proposed transformed HOG method.

Dataset	Cell size	Measurements	Our method (SD)	Baseline method (SD)
DS-Detect	20	ACC	86.3% (1.4)	76.8% (1.2)
		SEN	89.4% (1.9)	84.1% (1.9)
		SPE	80.5% (2.5)	63.7% (2.4)
		AUC	0.921 (0.007)	0.846 (0.013)

**TABLE 2:** Comparison of the glioma grading performance evaluated by 10-fold cross-validation between the baseline concatenating HOG method and our proposed transformed HOG method.

Dataset	Cell size	Measurements	Our method (SD)	Baseline method (SD)
DS-Grade	18	ACC	76.3% (2.4)	72.0% (2.3)
		SEN	83.7% (3.2)	72.5% (3.4)
		SPE	68.7% (2.7)	71.6% (2.8)
		AUC	0.806 (0.022)	0.777 (0.021)

For summarizing the performance of our proposed classification method, we showed the confusion matrix for glioma detection task and glioma grading task in Tables 3, 4, respectively.

**TABLE 3:** Confusion matrix for glioma detection task.

		Predicted class	
		
		glioma	non-glioma
Actual class	glioma	TP = 56	FN = 6
	non-glioma	FP = 6	TN = 31

**TABLE 4:** Confusion matrix for glioma grading task.

		Predicted class	
		
		GBM	LGG
Actual class	GBM	TP = 62	FN = 14
	LGG	FP = 19	TN = 39

## Discussion

As an advanced diagnostic imaging technology, brain MRI can provide more objective and reliable evidence for tumor detection and grade evaluation with invasive procedure. In the present study, a two-phase classification framework based on HOG features was developed for supporting clinical diagnosis of brain tumors such as gliomas. We applied the framework to two different tasks including identifying patients with gliomas from healthy controls and differentiating between GBM (WHO grade IV) and LGG (WHO grade II and III). In clinic, the glioma detection task is the first step for the subsequent grade differentiation. The performance for glioma detection task achieved an accuracy of 86.3%, a sensitivity of 89.4%, a specificity of 80.5%, and an AUC of 0.921. The results may be good enough to provide diagnostic suggestions to physicians. By comparison, the glioma grading performance achieved an accuracy of 76.3%, a sensitivity of 83.7%, a specificity of 68.7%, and an AUC of 0.806. The reason may be that the differences between healthy brains and diseased brains are more significant than those between GBM and LGG. Furthermore, the heterogeneous composition of aggressive cellular tissues may also cause misdiagnoses. Although machine learning techniques have been widely used in prior tumor grading studies, it is striking that most of these previous studies used the state-of-the-art radiomics or deep learning methods (Skogen et al., 2016; Hsieh et al., 2017a; Yang et al., 2018; Zhuge et al., 2020). Some of these approaches obtained grade classification accuracy over 90%. However, these methods are more dependent on stable and reproducible segmentation of the ROI, and a large amount of high-dimensional feature extraction and evaluation. Although our two-phase classification framework did not achieve much high performance on tumor grading, we directly extracted gradient features from the MRI images without much computational complexity. And our method did not depend on tumor segmentation by physicians or algorithms. Thus, our automated diagnostic tool may be more appropriate for clinical usage.

The first contribution of our work is that we have applied the computer vision techniques, e.g., HOG descriptor, to the analysis of MRI medical images. HOG and other feature descriptors such as SIFT are commonly used in object detection task such as human face identification. Compared to SIFT that only captures some salient key feature points, HOG describes the gradient change for each pixel (voxel for 3D HOG), so HOG features are good at depicting small or subtle changes within brain. Since the gliomas can cause local occupying effect, some structural changes will occur in the brain, which can lead to abnormal intensity-based gradient changes on the MRI images of patients with gliomas compared to images of healthy brains. For different grades of gliomas, the gradient change pattern is also supposed to be different thus can be used as discriminating features to estimate the tumor grade.

The second contribution of our work is to propose the two-phase classification framework. In our framework, we calculated HOG feature on each local brain region that is called a “cell” in the algorithm. Instead of directly concatenating each HOG feature into one feature vector as what it is traditionally used in pedestrian detection, we analyzed each HOG feature independently using machine learning methods. Specifically, we applied the fuzzy clustering method on HOG features from the same position on an MRI image, which transformed each HOG feature into a membership probability related to diseased status. Then we can obtain a high-level semantic feature by concatenating the clustered result of each HOG feature representing the distribution pattern of diseased brain regions for the whole MRI image. On the one hand, this transformation between different feature space can indeed reduce the dimensionality of the final feature representation in order to avoid overfitting, which is especially necessary for 3D MRI images including many 2D slices and relatively small number of annotated medical images; on the other, it enables us to identify the tumor-related features that contribute most to the final classification result. We have adopted such region-independent analysis approach in our previous studies on diagnosis of Alzheimer’s disease, Parkinson’s disease, bipolar disorder and autism using MRI images (Chen et al., 2014, 2020). And the results showed that this approach analyzing local features independently could facilitate the identification of potential tumor regions in neurological diseases.

In addition to the brain tumor classification framework, we also developed a computer-aided diagnosis platform for brain tumor integrating different parts of the framework. Currently, this platform needs to manually transfer the original DICOM image data to the platform, which is not efficient and timely. Next, we plan to develop a network interface between the platform and the Picture Archiving and Communication Systems (PACS) of Zhongnan Hospital of Wuhan University. This interface can ensure an automatic imaging data transfer from PACS to the platform, which may improve the applicability of the platform in clinical settings.

In addition to the strengths discussed earlier, our study has several limitations. First, we used only T2-Flair sequences that can depict peritumoral edema with clear signals, while may be weak in demonstrating other tumor characteristics such as necrosis and angiogenesis module. In the future study, we plan to investigate the possible complementary power from other MRI sequences under routine preoperative protocol. Second, the DS-Grade dataset includes both grade II and III gliomas with three different histological cell types including astrocytoma, oligodendroglioma, oligoastrocytoma. Each of the glioma subtypes is considered to contain different MRI signature. And the heterogeneity within the DS-Grade dataset may account for the poorer model performance for differentiating GBM and LGG. So, it is necessary to collect sufficient data consisting of various glioma subtypes for model validation in the next study. Third, our study did not consider demographic information of the subjects (e.g., patient age), which may provide additional discriminatory value.

## Data Availability Statement

The raw data supporting the conclusions of this article will be made available by the authors, without undue reservation.

## Ethics Statement

The studies involving human participants were reviewed and approved by the Institutional Ethics Board of Zhongnan Hospital of Wuhan University. Written informed consent for participation was not required for this study in accordance with the national legislation and the institutional requirements.

## Author Contributions

TC and LL designed the study. TC implemented the algorithm. MY preprocessed the imaging data. ZY, FX, and HX gave critical suggestions. TC, MY, and LL drafted the manuscript. All authors contributed to the article and approved the submitted version.

## Conflict of Interest

The authors declare that the research was conducted in the absence of any commercial or financial relationships that could be construed as a potential conflict of interest.
